# COVID-19 outbreaks surveillance through text mining applied to electronic health records

**DOI:** 10.1186/s12879-024-09250-y

**Published:** 2024-03-28

**Authors:** Hermano Alexandre Lima Rocha, Erik Zarko Macêdo Solha, Vasco Furtado, Francion Linhares Justino, Lucas Arêa Leão Barreto, Ronaldo Guedes da Silva, Ítalo Martins de Oliveira, David Westfall Bates, Luciano Pamplona de Góes Cavalcanti, Antônio Silva Lima Neto, Erneson Alves de Oliveira

**Affiliations:** 1 Department of Community Health, Federal University of Ceará, Street Papi Júnior, 1223, 5th. Floor, Fortaleza, CE Brazil; 2 Postgraduate Program in Applied Informatics, University of Fortaleza, Fortaleza, CE 60811-905 Brazil; 3 Health Secretariat, Ceará State Government, Fortaleza, CE Brazil; 4 Harvard Medical School, Boston, MA USA; 5 School of Public Health of Ceará, Fortaleza, CE Brazil; 6 Faculty of Medicine, Christus University Center, Fortaleza, CE Brazil; 7 Laboratory of Data Science and Artificial Intelligence, University of Fortaleza, Fortaleza, Ceará, 60811-905 Brazil; 8 Professional Masters in City Science, University of Fortaleza, Fortaleza, Ceará, 60811-905 Brazil

**Keywords:** COVID-19, Disease outbreaks, Public Health Surveillance, Data Science, Epidemiology

## Abstract

**Background:**

The COVID-19 pandemic has caused significant disruptions to everyday life and has had social, political, and financial consequences that will persist for years. Several initiatives with intensive use of technology were quickly developed in this scenario. However, technologies that enhance epidemiological surveillance in contexts with low testing capacity and healthcare resources are scarce. Therefore, this study aims to address this gap by developing a data science model that uses routinely generated healthcare encounter records to detect possible new outbreaks early in real-time.

**Methods:**

We defined an epidemiological indicator that is a proxy for suspected cases of COVID-19 using the health records of Emergency Care Unit (ECU) patients and text mining techniques. The open-field dataset comprises 2,760,862 medical records from nine ECUs, where each record has information about the patient’s age, reported symptoms, and the time and date of admission. We also used a dataset where 1,026,804 cases of COVID-19 were officially confirmed. The records range from January 2020 to May 2022. Sample cross-correlation between two finite stochastic time series was used to evaluate the models.

**Results:**

For patients with age ≥18 years, we find time-lag ($\tau_c$) = 72 days and cross-correlation (${\widehat p}_{ij}$) ~0.82, $\tau_c$ = 25 days and ${\widehat p}_{ij}$ ~0.93, and $\tau_c$ = 17 days and ${\widehat p}_{ij}$ ~0.88 for the first, second, and third waves, respectively.

**Conclusions:**

In conclusion, the developed model can aid in the early detection of signs of possible new COVID-19 outbreaks, weeks before traditional surveillance systems, thereby anticipating in initiating preventive and control actions in public health with a higher likelihood of success.

## Introduction

The COVID-19 pandemic has had a profound impact on the world. It is considered one of humanity’s worst crises since World War II [1]. The pandemic has caused significant disruptions to everyday life and has had social, political, and financial consequences that will persist for years and decades. The global economy has suffered due to the pandemic, leading to governments implementing measures to mitigate the spread of the virus and revitalize their economies. The pandemic has also affected individuals’ physical and mental health, with healthcare workers experiencing psychological distress and individuals with pre-existing conditions like fibromyalgia experiencing worsened symptoms [2]. The socioeconomic implications of the pandemic have been far-reaching, affecting various aspects of the world economy [3]. Both developed and developing countries presented insufficient health facilities to tackle this emergency and struggled with socioeconomic issues, making it harder to implement social distancing measures [4]. So far, over 37 million cases and more than 700,000 deaths have been registered in Brazil [5, 6].

This pandemic pressured healthcare systems worldwide, both in terms of resources for treatment [7], vaccine administration [8], and case monitoring capacity [9], leading to a significant investment in solutions that could optimize the response to the pandemic. Several initiatives with intensive use of technology were quickly developed in this scenario, ranging from improvements in medical equipment for patient respiratory ventilation [10] to the extensive use of telemedicine [11] and the use of Artificial Intelligence (AI) models to estimate patient outcomes [12, 13]. Among the advancements, the epidemiological surveillance of cases also progressed.

Epidemiological surveillance can be understood as “the systematic and continuous collection, analysis, interpretation, and dissemination of data related to a health event to take action” [14]. Surveillance is essential to public health management, and public managers should make evidence-based decisions whenever possible [15]. Since the beginning of the COVID-19 pandemic, global interest in real-time monitoring of the number of reported cases and deaths and, more importantly, their trend and prognosis, which directly impacted the lives of all people, revealed the relevance of robust epidemiological surveillance systems [5]. However, most surveillance systems currently in operation rely on active reporting of cases, whether outpatient, in hospitals, or deaths, surveillance through sentinel networks, laboratory test results, and active surveillance through interviews conducted by surveillance teams [14]. These systems depend on patients reaching the healthcare system, either due to suspicion of COVID-19 by a healthcare professional or to undergo testing for the disease. This dependency can limit the surveillance of new cases and potential outbreaks.

Despite intensive technology use in various healthcare sectors, and, more accelerated during the pandemic, developing technologies that enhance epidemiological surveillance with better case detection, especially in contexts with low testing capacity and healthcare resources, is scarce and in the prototype phase. For example, publications on AI tools to surveillance new and suspected COVID-19 cases using routinely collected data from health records are scarce [16]. Therefore, this study aims to address this gap by developing a data science model that uses routinely generated healthcare encounter records to detect possible new outbreaks of COVID-19 in real-time.

## Methods

### The Model

We define an epidemiological indicator that is a proxy for suspected cases of COVID-19 using the dataset of Emergency Care Unit (ECU) patients (see Datasets) and text mining techniques [17]. By Text Mining, we mean a set of techniques for the automatic extraction of non-trivial information from digital texts. Here, we search for two consecutive patterns in each entry of our reported symptoms field. The first pattern is the string matching for the word “dyspnea’’ (“*dispneia*’’, in Portuguese) and its synonyms: “shortage of breath’’ (“*falta de ar*’’) and “respiratory discomfort’’ (“*desconforto respitatório*’’). Once we find any of these terms, we search for the second pattern, the string matching for the words “cough’’ (“*tosse*’’, in Portuguese) and “fever’’ (“*febre*’’). If we find at least one of them, we consider this patient a suspected case of COVID-19. Finally, the proposed indicator is the daily number of suspected cases. We chose Python [18] as the programming language for the entire study. In this context, we used the Pandas [19] and Unidecode [20] modules for data cleaning, while the Matplotlib [21] module was used to generate all figures.

### Datasets

#### ECU patients

The secretary of health of the state of Ceará compiled and made available an anonymized dataset (provided in Comma Separated Values - .csv - format) of ECU patients from Fortaleza, Ceará, Northeast of Brazil (see Data Availability). The Brazilian ECU (“*Unidades de Pronto Atendimento*’’, in Portuguese, or UPA’s, for short) are emergency care units that represented the base of the public health system during the pandemic in Brazil. The dataset ranges from January 2020 to May 2022 and comprises 2,760,862 medical records from nine ECUs, where each record has information about the patient’s age, reported symptoms, and the time and date of admission. We highlight the fact that health professionals are encouraged to not abbreviate medical terms when filling in the entries of the reported symptoms.

#### Confirmed cases of COVID-19

The dataset of confirmed cases of COVID-19 from the city of Fortaleza, Ceará, Brazil, is available on the IntegraSUS platform [22]. We have collected a dataset corresponding to the period from January 2020 to May 2022, where 1,026,804 cases of COVID-19 were officially confirmed. Here, each record is a confirmed case of COVID-19 with information about the patient’s age and record date. This dataset is also formatted in .csv.

#### Sample cross-correlation

The sample cross-correlation between two finite stochastic time series, $X_i=\left\{X_i^t\right\}_{t=1}^N$ and $X_j=\left\{X_j^t\right\}_{t=1}^N$ is calculated as follows [23]:

1 $${\widehat P}_{i,j}=\frac{{\sum_{t=1}^N}\left[\left(X_i^t-{\overline X}_i\right)\left(X_j^t-{\overline X}_j\right)\right]}{\sqrt{{\sum_{t=1}^N}\left(X_i^t-{\overline X}_i\right)^2}\sqrt{{\sum_{t=1}^N\left(X_j^t-{\overline X}_j\right)}^2}}$$

where $N$ is the number of observations and ${\overline X}_i=\frac1N\overset N{\underset{t=1}{\sum X_i^t}}$  is the temporal mean of $X_i^t$. The sample cross-correlation ${\widehat P}_{ij}$ is a dimensionless similarity measure that ranges from, a perfect negative correlation, to, a perfect positive correlation. A negative correlation means that if $X_i$ tends to increase, $X_j$ tends to decrease. A positive correlation means the opposite, i.e., if $X_i$ tends to increase, $X_j$ also tends to increase. This measure is similar to the Pearson correlation coefficient [24].

#### The characteristic time lag $\tau_c$ 

Another important measure in Time Series Analysis is the time lag [25]. Given the iterative process with periodic boundary conditions that keep $X_i$ stationary while $X_j$ is slid in relation to $X_i$ every time unit, the time lag $\tau$  between $X_i$ and $X_j$  of each iteration can be measured through the accumulated number of slidings. Therefore, the characteristic time lag $\tau_c$ is defined as the value of $\tau$  associated with the maximum value of ${\widehat P}_{ij}$ during such a process.

#### Ethics approval

The Research Ethics Committee of the Unichristus University Center approved the survey. All methods were carried out in accordance with relevant guidelines and regulations. If applicable, informed consent was obtained from all subjects and/or their legal guardian(s).

## Results

**Fig. 1:**
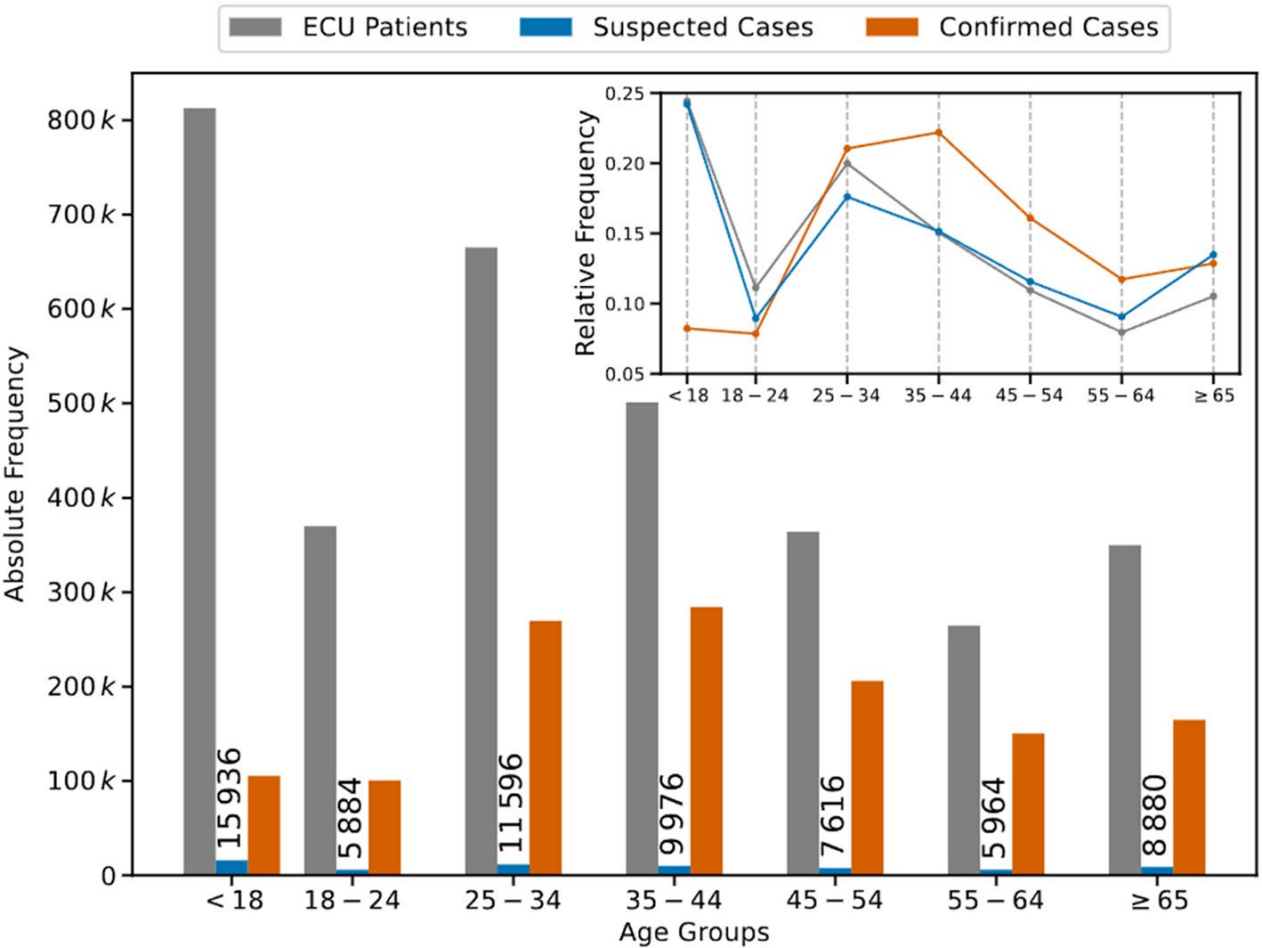
Frequencies stratified by age for ECU patients and confirmed cases of COVID-19 datasets. The bar chart shows the absolute frequency of age groups for both datasets (in gray and orange). Further, we also show the suspected cases of COVID-19 (in blue), estimated by the proposed model from the ECU patients dataset. The line chart (inset) shows the relative frequency of age groups for these quantities. We note the similarity between the relative frequencies of ECU patients and suspected cases, suggesting good representativeness of the suspected cases of COVID-19 compared to the ECU patients dataset

Figure 1 shows the absolute frequency of the age groups for the ECU patients, suspected cases of COVID-19, and confirmed cases of COVID-19. The frequency of ECU patients for the 18 age group is the highest among all strata. The same is not observed for the frequency of confirmed cases, where the highest value is found for the 35-44 age group. We show the relative frequency of the three quantities in the inset since the absolute frequency of the suspected cases is much lower than the others. The relative frequencies of ECU patients and suspected cases follow a similar behavior for patients ages 18 years, leading us to conclude that the frequency of suspected cases is not biased to such age groups. Therefore, we left the age stratum of patients ages <18 years out of the main analysis since children are more susceptible to seasonal respiratory diseases.

Table 1 summarizes the string matching found in the ECU patients dataset. For pattern 1, we count 101,523 for “dyspnea”, 6,999 for “respiratory discomfort”', and 44,916 for “shortage of breath”, as shown in the first and second columns. For each string matching in pattern 1, we also count the words “cough”, “fever”, and both as described in the third and fourth columns. The total number of suspected cases estimated by the proposed model is 75,289 (~7% of 1,026,804, the total number of confirmed cases). The most frequent combined pattern is “dyspnea” and “cough”, corresponding to 30,828 (~41%) suspected cases. Furthermore, 90,432 patients' records match only pattern 1, indicated by “-” in the third column, which is not counted as suspected cases. We emphasize that the frequencies of the correctly spelled words are notably higher than the misspelled ones for all symptoms, which makes us believe that these variations would not change our results.

**Table 1:** Counting of the string matching. The first and second columns are related to the first pattern, which consists of the string matching for the dyspnea synonyms (“Dyspnea”, “Respiratory Discomfort”, and “Shortage of Breath”) found in the reported symptoms field from the dataset of ECU patients. The following two columns are related to the second pattern, which is only searched if the first one is found. The second pattern is associated with the string matching of the words “Cough”, “fever”, or both, where the absence of matching is represented by “-”. The most frequent combined pattern is “Dyspnea” and “Cough”, corresponding to 30,828 (~41%) suspected cases, while the total number of suspected cases is 75,289 (~7$% of 1,026,804, the total number of confirmed cases) in the studied period, calculated by the sum of all string matchings for the first and second patterns.

Pattern 1	Total	Pattern 2	Total Combined
Dyspnea	101,523	Cough	30,828
		Fever	9,137
		Cough & Fever	12,701
		-	48,857
Respiratory Discomfort	6,999	Cough	1,907
		Fever	648
		Cough & Fever	795
		-	3,649
Shortage of Breath	44,916	Cough	10,373
		Fever	4,414
		Cough & Fever	4,486
		-	25,643
		Total Suspected Cases = 75,289	

**Fig. 2:**
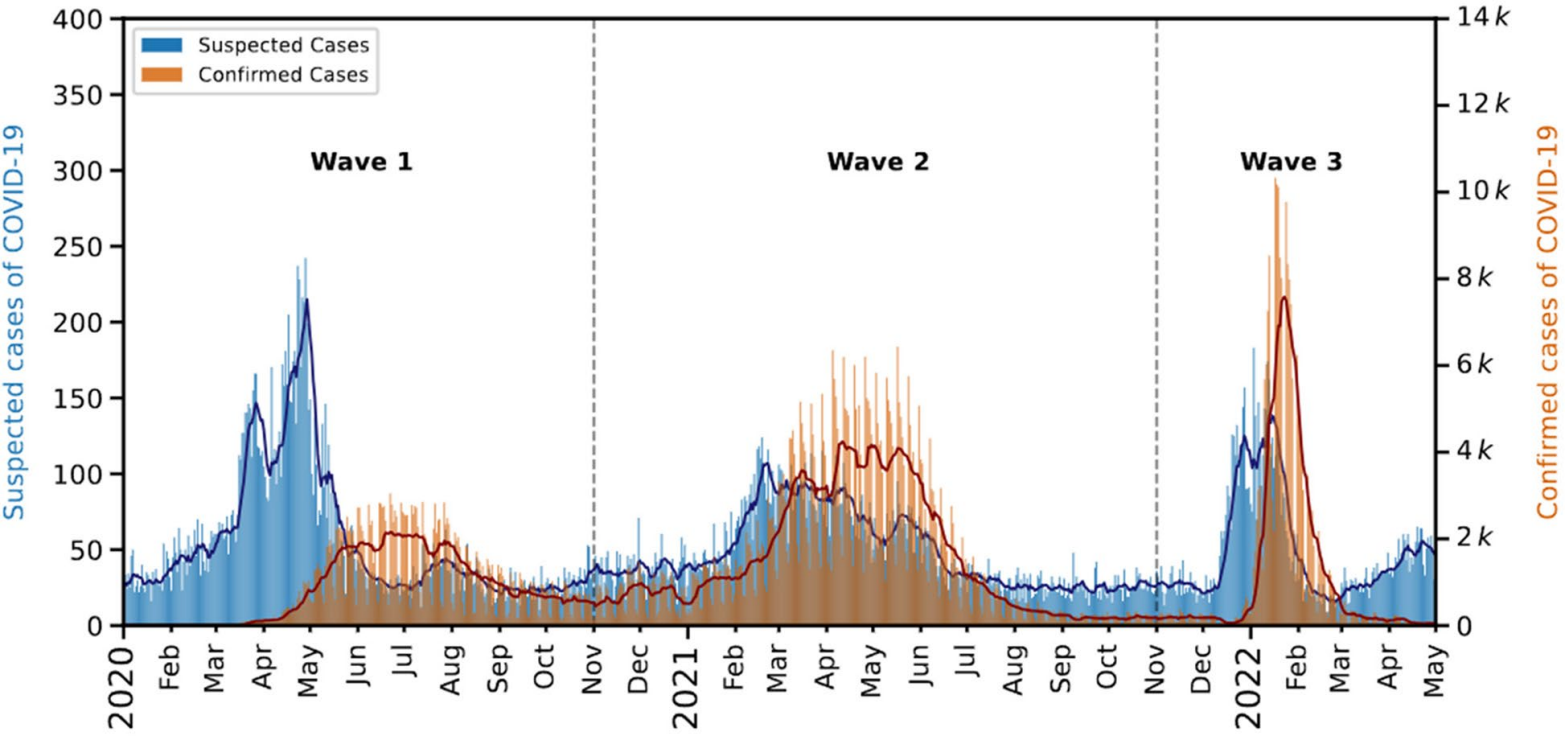
Time series of suspected and confirmed cases of COVID-19. The bars represent the daily numbers of suspected (in blue) and confirmed (in orange) cases of COVID-19. Here, we consider only cases with ages 18 years. The solid lines represent moving averages with windows of 7 days for both quantities. The time series are divided into three epidemiological waves: The first occurred between January 2020 and November 2020, the second between November 2020 and November 2021, and the third between November 2021 and May 2022. We note that the time series of suspected cases of COVID-19 is delayed in comparison to the time series of confirmed cases of COVID-19, endorsing the idea that it is possible to early detect COVID-19 waves using only the patient's reported symptoms

We show the time series of confirmed and suspected cases of COVID-19 in Fig. 2. The studied period encloses the first three epidemiological waves in Fortaleza. Here, the suspected and confirmed cases for patients with age 18 years are depicted in blue and red, respectively. The bars are the daily number of cases, and the solid lines are the weekly moving averages. Moving averages smooth the fluctuations of daily data, leading to a better visualization of time series even in cases with the presence of holidays and weekends in the studied period. Furthermore, the time interval of 7 days is a common choice for moving averages in Epidemiology since it is a value greater than 3 days (to attenuate the fluctuation due to missing data on long weekends) and matches the natural periodicity of the pace of the societies. The suspected cases curve seems to precede the confirmed cases' peaks for each wave.

**Fig. 3:**
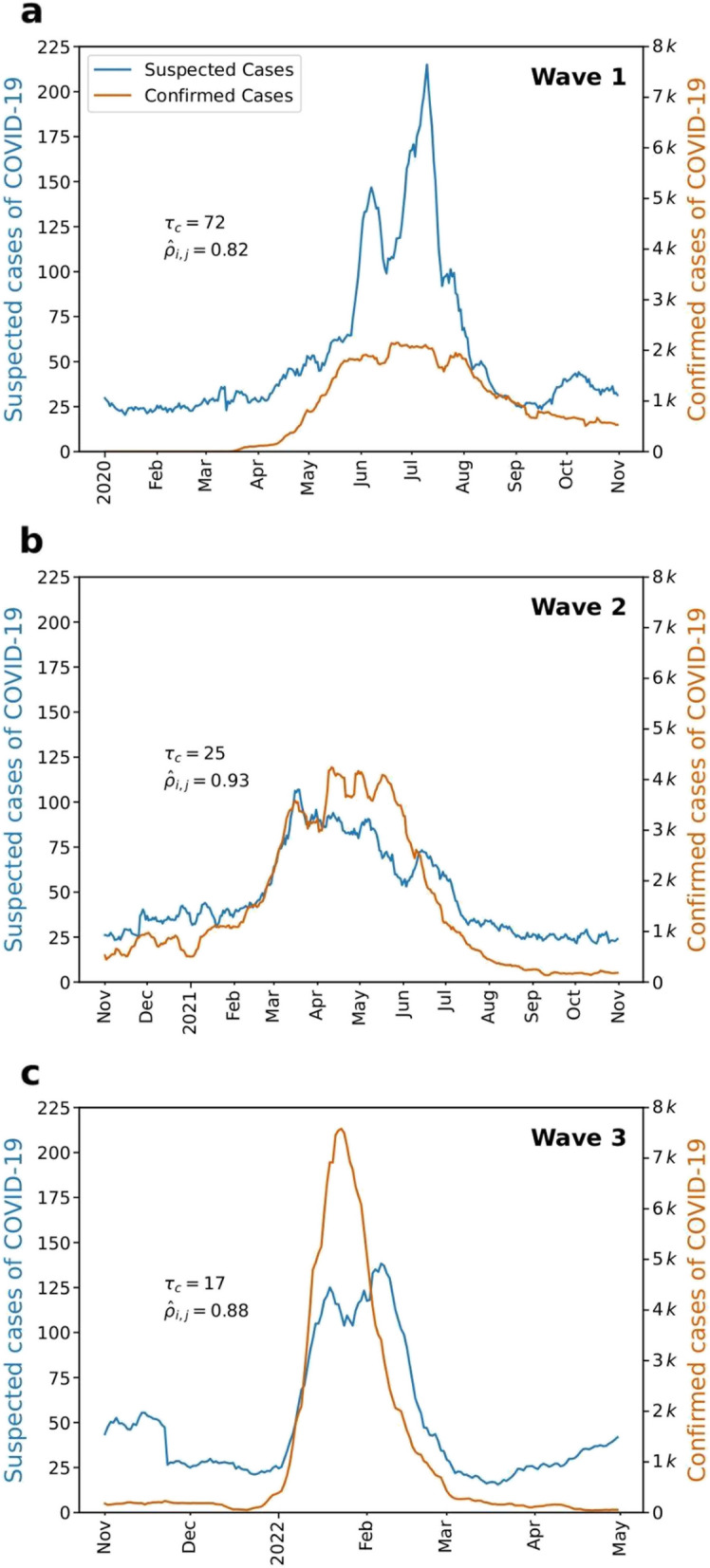
The shifted time series of suspected cases of COVID-19 for patients with ages 18 years. For each epidemiological wave, we shift the time series of suspected cases of COVID-19 (in blue) by the characteristic time lag , the time lag associated with the maximum value of the sample cross-correlation , and compare it to the time series of confirmed cases of COVID-19 (in orange). (a)-(c) We find = 72 days and ~0.82, = 25 days and ~0.93, and = 17 days and ~0.88 for the first, second, and third waves, respectively. Despite the high values of in all waves, the last two waves represent a more trustful value of since there was a well-known lack of tests in the first wave of COVID-19

As shown in Fig. 3, we compare the time series of confirmed cases with the time series of suspected cases shifted by for each COVID-19 wave. For patients with age 18 years, we find = 72 days and ~0.82, = 25 days and ~0.93, and = 17 days and ~0.88 for the first, second, and third waves, respectively.

**Fig. 4:**
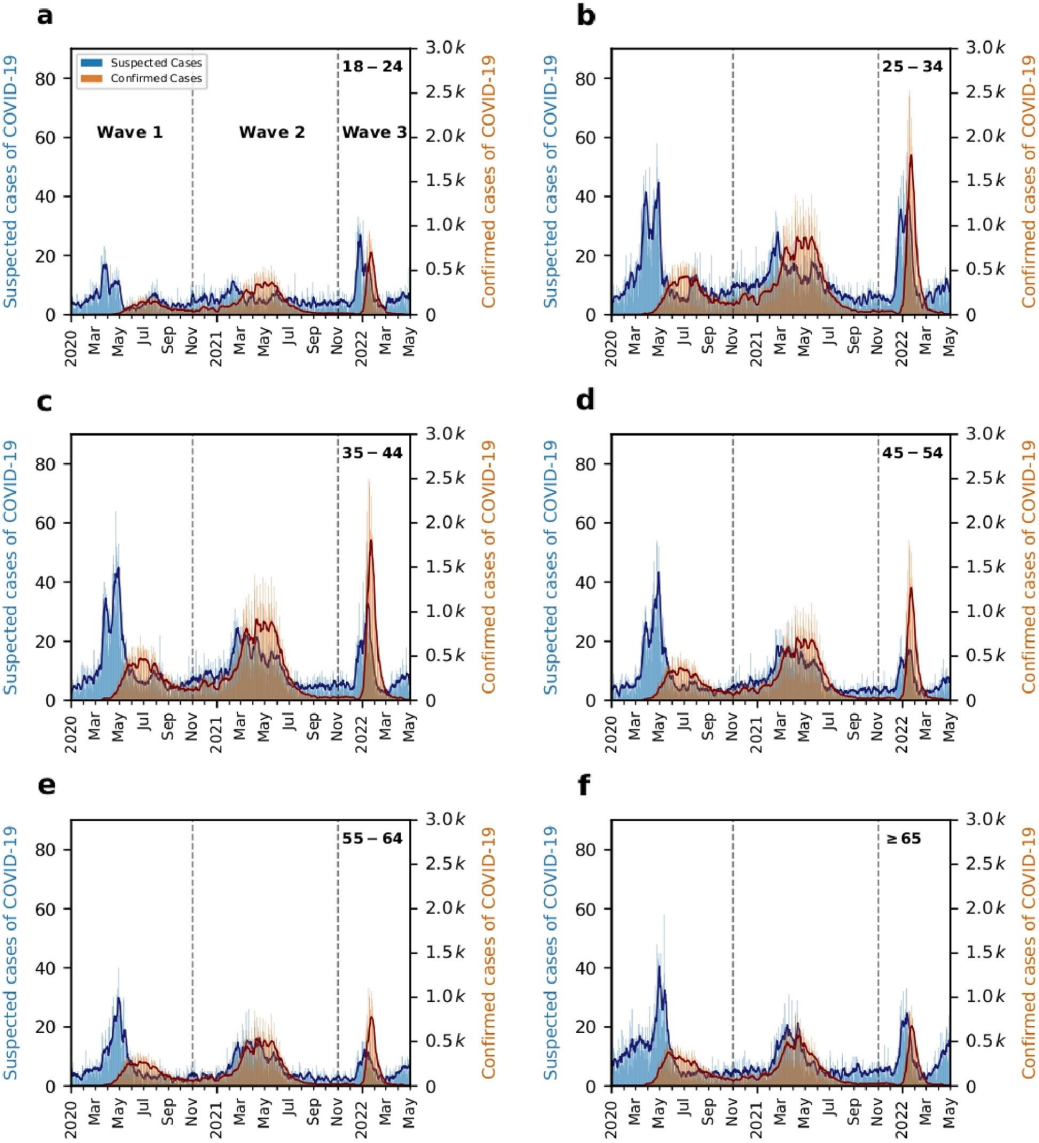
Stratified time series of suspected and confirmed cases of COVID-19. The bars represent the daily numbers of suspected (in blue) and confirmed (in orange) cases of COVID-19. The solid lines represent moving averages with windows of 7 days for both quantities. We show the time series of suspected cases of COVID-19 stratified by the following age groups: patients with ages (**a**) 18-24 years, (**b**) 25-34 years, (**c**) 35-44 years, (**d**) 45-54 years, (**e**) 55-64 years, and (**f**) 65 years. We leave the age stratum of patients with age <18 years out of the main analysis due to its particular behavior likely related to seasonal respiratory diseases in children. We also emphasize that, in all remaining strata, the time series of suspected cases of COVID-19 are delayed in comparison to the time series of confirmed cases of COVID-19, endorsing, even more, the idea that it is possible to early detect COVID-19 waves using only the patient's reported symptoms

Figure 4 shows the time series of confirmed and suspected cases of COVID-19 stratified by six age groups, which are: 18-24, 25-34, 35-44, 45-54, 55-64, and 65 years. We find that the time series of suspected cases is delayed compared to the time series of confirmed cases, showing above 0.59 in all scenarios. We summarize all values of and in Table 2. (TABLE 2) Such results suggest that the daily number of suspected cases of COVID-19, i.e., the proposed epidemiological indicator, shown in Fig. 3, is indeed not biased by age.

**Table 2:** Characteristic time lag and its associated sample cross-correlation stratified by age for each epidemiological wave of COVID-19. The rows correspond to the waves, and the columns correspond to age groups (18-24, 25-34, 35-44, 45-54, 55-64, and65 years), except the last one (Total) that combines all strata. We find that the values of for the first wave are greater than those observed in the second and third waves, probably, due to the lack of COVID-19 tests at that time. The ranges between 0.59 and 0.95 in all strata. We emphasize that, for a given wave, the values of are quite similar among the strata, suggesting that our results are unbiased in relation to age groups

	Age Groups													
	18-24		25-34		35-44		45-54		55-64		≥65		TOTAL	
	$\tau_c$	${\widehat p}_{i,j}$	$\tau_c$	${\widehat p}_{i,j}$	$\tau_c$	${\widehat p}_{i,j}$	$\tau_c$	${\widehat p}_{i,j}$	$\tau_c$	${\widehat p}_{i,j}$	$\tau_c$	${\widehat p}_{i,j}$	$\tau_c$	${\widehat p}_{i,j}$
Wave 1	96	0.69	92	0.77	67	0.80	66	0.80	61	0.81	73	0.71	72	0.82
Wave 2	85	0.59	26	0.77	23	0.90	22	0.95	22	0.94	18	0.90	25	0.93
Wave 3	25	0.91	17	0.88	12	0.89	11	0.82	16	0.82	18	0.86	17	0.88

## Discussion

This study introduces a text mining model that uses routinely generated healthcare records data to detect possible new outbreaks of COVID-19 cases in real time, thereby assisting in public health decision-making. Although we developed the model to access real-time data, both datasets used here were historical records. Actually, as a counterpart for the availability of the dataset, we provided our model to the secretary of health of the state of Ceará (SESA). Therefore, they can update the time series of suspected cases whenever needed (hourly, daily, weekly, etc.). Our system was able to detect possible outbreaks around 15 days before the usual notification systems (active and laboratory notification). The system was made available through the integraSUS platform, an electronic platform already existing in the health department and widely used and known by managers for managing actions, which can be seen at https://integrasus.saude.ce.gov.br/#/home. In this system, the final output of the model, the graph of suspected cases with the flags of possible outbreaks, is visible in real time, and is displayed on large televisions in the state’s health indicator situation rooms, which health managers have access. This data is also shared with other bodies, such as the state public ministry, for joint monitoring. The correlation between the modeled and actual data was 0.82, 0.93, and 0.88 in waves one, two, and three, respectively, considering patients aged 18 years or above.

Real-time analysis of epidemiological data is crucial for guiding immediate or planned interventions to promptly address an epidemiological problem as it arises [14]. The data used by the system we developed is generated in real-time during patient care in emergency units, making it immediately available for analysis. Logically, the volume of information generated would make it impossible to manually generate indicators from this mass of data, which is why using artificial intelligence in this context is essential. Using the model, data that would typically be lost provides valuable and timely information for health system managers. In addition, our system utilizes resources and technologies routinely available in healthcare units in Brazil. Also, it can operate in real-time even with data collected from mobile units that reach deep into the heart of the Amazon rainforest, for example. A similar idea was used in The Public Health England Emergency Department Syndromic Surveillance System (EDSSS), which carried out near real-time public health surveillance of emergency department (ED) attendances across England [26]. However, this system didn’t use NLP, it only counted structured data. Also, a recent systematic review showed advances in the use of natural language processing for surveillance, but it was used in informal internet texts [27]. Finally, a study carried out in Israel used a similar technique to detect Covid cases, with specificity found to be 81.5%. However, 78 cases were used to verify the model’s accuracy at an individual level, not a curve level [28]. Therefore, the initiative presented in our article is unique in the current context of epidemiological surveillance.

Our results showed signs suggestive of an outbreak up to 71 days before the same signs were identified by confirmatory testing for the disease. This result was demonstrated in the first wave when the availability of tests was meager, suggesting that a COVID-19 outbreak could be detected earlier by the proposed model than by the testing, especially for emerging and re-emerging diseases or for testing shortages. The developed system identified the likely occurrence of new waves an average of 3 weeks before detection based on official COVID-19 confirmed cases data. This information can be crucial in conjunction with other data to assist in initiating measures to prevent large disease outbreaks.

Identifying new suspected or confirmed cases of COVID-19 is essential for effective preventive public health interventions aimed at reducing the spread of the disease and containing it, such as social distancing decrees, to prevent future outbreaks [15, 29]. Despite efforts to enhance epidemiological surveillance, developing countries still struggle to identify, diagnose accurately, and report these infectious diseases [30]. For instance, access to free testing in these countries is still limited. As a result, patients with mild or moderate cases may not undergo testing, leading to underestimating the number of cases. With the advancement of vaccination coverage, more patients present only mild or moderate cases, are treated symptomatically in emergency care settings, and may not be tested. These patients can be missed by surveillance systems that rely solely on testing or active reporting from healthcare facilities. In this regard, the model we developed using clinical care data can help identify cases of patients whom traditional surveillance systems would not identify. This improved detection capacity can be observed when comparing our results with the results from the traditional surveillance system used in Brazil. During the first wave, for example, the healthcare system did not have sufficient tests or infrastructure for mass testing of COVID-19 cases across the vast dimensions of the country [31]. Therefore, our system identified significantly more cases than the official system.

Despite the advances in controlling infectious diseases worldwide, diseases such as measles, polio, and dengue remain concerns that afflict healthcare systems. For instance, many countries in the Americas have recently reported measles cases, including Brazil, Venezuela, Canada, the United States, Mexico, Peru, and Argentina [32]. In 2018, an outbreak that started in the Northern region of Brazil resulted in cases spreading to multiple states, including indigenous communities. Consequently, Brazil lost its status as a measles-free country [33]. It is evident, therefore, that infectious diseases continue to generate outbreaks, epidemics, and health impacts on society. The model we developed can be adapted for the surveillance of other diseases with significant impact, and we have been working on adapting it for the surveillance of dengue cases, in a scenario of simultaneous transmission of other arboviruses.

Finally, our string-matching model has some limitations related to the following two factors. First, the proposed model is reliant on the medical care records quality. Misspelling and missing values, like patient’s age, impact the results. Second, the model does not use health measurements and medical exams. Adding this objective data to the patient’s reported symptoms could make this model even more robust.

## Conclusion

In conclusion, the developed model can aid in the early detection of signs of possible new COVID-19 outbreaks several days before traditional surveillance systems, thereby assisting in initiating preventive and control actions in public health with a higher likelihood of success.

## Data Availability

The data underlying this article were provided by the Secretary of Health of the State of Ceara - Brazil (SESA) by permission after the Ethical Review Board of Brazil approval. Data will be shared on request to the corresponding author with permission of SESA.

## Abbreviations

ECU: Emergence Care Unit
