# Correction: COVID-19 outbreaks surveillance through text mining applied to electronic health records

**DOI:** 10.1186/s12879-024-09292-2

**Published:** 2024-04-11

**Authors:** Hermano Alexandre Lima Rocha, Erik Zarko Macêdo Solha, Vasco Furtado, Francion Linhares Justino, Lucas Arêa Leão Barreto, Ronaldo Guedes da Silva, Ítalo Martins de Oliveira, David Westfall Bates, Luciano Pamplona de Góes Cavalcanti, Antônio Silva Lima Neto, Erneson Alves de Oliveira

**Affiliations:** 1 Department of Community Health, Federal University of Ceará, Street Papi Júnior, 1223, 5Th. Floor, Fortaleza, CE Brazil; 2 Postgraduate Program in Applied Informatics, University of Fortaleza, Fortaleza, CE 60811-905 Brazil; 3 Health Secretariat, Ceará State Government, Fortaleza, CE Brazil; 4 Harvard Medical School, Boston, MA USA; 5 School of Public Health of Ceará, Fortaleza, CE Brazil; 6 Faculty of Medicine, Christus University Center, Fortaleza, CE Brazil; 7 Laboratory of Data Science and Artifcial Intelligence, University of Fortaleza, Fortaleza, Ceará 60811-905 Brazil; 8 Professional Masters in City Science, University of Fortaleza, Fortaleza, Ceará 60811-905 Brazil

**Correction** **:** **BMC Infect Dis 24, 359 (2024)**

**https://doi.org/10.1186/s12879-024-09250-y**

Following publication of the original article [1], we have been notified that the Results section of the Abstract was missing the mathematical symbols.

Originally published Results:


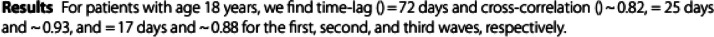


Corrected Results:


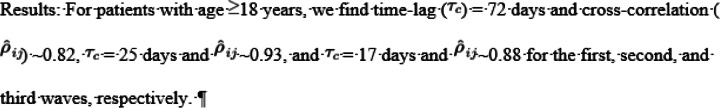


The original article has been corrected.
